# Optimized but Not Maximized Cue Integration for 3D Visual Perception

**DOI:** 10.1523/ENEURO.0411-19.2019

**Published:** 2020-01-02

**Authors:** Ting-Yu Chang, Lowell Thompson, Raymond Doudlah, Byounghoon Kim, Adhira Sunkara, Ari Rosenberg

**Affiliations:** 1 Department of Neuroscience, School of Medicine and Public Health, University of Wisconsin–Madison, Madison, WI 53705; 2 Department of Surgery, School of Medicine and Public Health, University of Wisconsin–Madison, Madison, WI 53705

**Keywords:** 3D visual perception, canonical computations, divisive normalization, optimal cue integration, perspective, stereoscopic

## Abstract

Reconstructing three-dimensional (3D) scenes from two-dimensional (2D) retinal images is an ill-posed problem. Despite this, 3D perception of the world based on 2D retinal images is seemingly accurate and precise. The integration of distinct visual cues is essential for robust 3D perception in humans, but it is unclear whether this is true for non-human primates (NHPs). Here, we assessed 3D perception in macaque monkeys using a planar surface orientation discrimination task. Perception was accurate across a wide range of spatial poses (orientations and distances), but precision was highly dependent on the plane’s pose. The monkeys achieved robust 3D perception by dynamically reweighting the integration of stereoscopic and perspective cues according to their pose-dependent reliabilities. Errors in performance could be explained by a prior resembling the 3D orientation statistics of natural scenes. We used neural network simulations based on 3D orientation-selective neurons recorded from the same monkeys to assess how neural computation might constrain perception. The perceptual data were consistent with a model in which the responses of two independent neuronal populations representing stereoscopic cues and perspective cues (with perspective signals from the two eyes combined using nonlinear canonical computations) were optimally integrated through linear summation. Perception of combined-cue stimuli was optimal given this architecture. However, an alternative architecture in which stereoscopic cues, left eye perspective cues, and right eye perspective cues were represented by three independent populations yielded two times greater precision than the monkeys. This result suggests that, due to canonical computations, cue integration for 3D perception is optimized but not maximized.

## Abstract (precis)

Reconstructing three-dimensional (3D) scenes from two-dimensional (2D) retinal images is an ill-posed problem. Despite this, 3D perception of the world based on 2D retinal images is seemingly accurate and precise. The integration of distinct visual cues is essential for robust 3D perception in humans, but it is unclear whether this is true for non-human primates (NHPs). Here, we assessed 3D perception in macaque monkeys using a planar surface orientation discrimination task.

## Significance Statement

Our eyes sense two-dimensional (2D) projections of the world, like a movie on a screen, but we perceive the world as three-dimensional (3D). Here, we show that non-human primates (NHPs), like humans, achieve more precise 3D vision by perceptually integrating distinct 3D cues. We also present evidence that perception is influenced by 3D natural scene statistics, and that priors over 3D orientation are subjectively encoded. Using simulations, we examine how neural computation can constrain 3D perception and estimate that perception is half as precise as theoretically possible. Our findings suggest that the concurrence of multiple canonical computations simultaneously optimizes and curbs 3D visual perception, and highlight that what constitutes optimal task performance depends on the underlying neural architecture.

## Introduction

**Figure 1.**
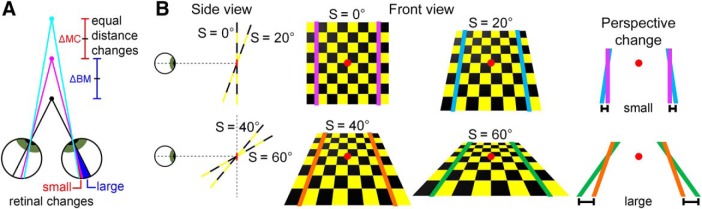
3D cue reliabilities depend on object pose. ***A***, Stereoscopic cue reliability decreases with distance. Equivalent changes in object distance produce smaller retinal image changes at greater distances. This is illustrated with an observer fixating the black dot. The distance between black and magenta dots (ΔBM) is equal to the distance between magenta and cyan dots (ΔMC), but the retinal change is larger for ΔBM than ΔMC. ***B***, The reliability of perspective cues increases with orientation-in-depth (slant). Equivalent slant changes produce larger changes in the rate at which parallel lines converge in the 2D projection at larger base slants. This is illustrated with a checkerboard rotated about the horizontal axis passing through the red dot. Colored lines are parallel in the world. A 20° slant (S) rotation produces a smaller perspective change between 0° and 20° (top row) than between 40° and 60° (bottom row).

Three-dimensional (3D) visual perception is a significant achievement of the primate brain (Barton, 2004). Because eyes sense two-dimensional (2D) projections of the world, 3D structure must be estimated. Creating 3D percepts from 2D images is a nonlinear optimization problem plagued by ambiguities and noise (Hartley and Zisserman, 2003). Studies with humans have shown that integrating distinct visual cues resolves ambiguities and improves 3D estimates (Knill and Saunders, 2003; Hillis et al., 2004; Welchman et al., 2005; Preston et al., 2009; Murphy et al., 2013; Rideaux and Welchman, 2018). In particular, stereoscopic and perspective cues have prominent roles in human 3D vision (Stevens, 1981; Howard and Rogers, 1995; Knill, 1998a). The reliability of these cues depends on an object’s spatial pose (position and orientation). Stereoscopic cue reliability decreases with distance from the observer (Fig. 1*A*). Perspective cue reliability increases with orientation-in-depth (slant; Fig. 1*B*). Humans perceptually integrate these cues near-optimally, with weights that match the reliabilities. Although non-human primates (NHPs) are an essential model system for studying the neural basis of 3D vision, surprisingly little is known about how they perceptually integrate these cues.

Here, we quantified 3D perception in NHPs using an eight-alternative forced choice (8AFC) surface orientation discrimination task. The contributions of stereoscopic and perspective cues to perception were assessed by presenting cue-isolated and combined-cue stimuli. With stereoscopic cues, performance decreased with distance from the fixation plane, consistent with the geometry of stereovision and the physiology of stereopsis (Howard and Rogers, 1995; Cumming and DeAngelis, 2001; Prince et al., 2002). With both cues, performance increased with slant. The perception of combined-cue stimuli was consistent with an optimal integration strategy (Murray and Morgenstern, 2010; Landy et al., 2011). Moreover, errors in perception could be explained by a prior resembling the 3D orientation statistics of natural scenes (Adams et al., 2016; Burge et al., 2016).

It is also unclear how the architecture of the visual system and circuit-level computations constrain 3D perception. The neuronal implementation of optimal cue integration is theoretically linear (Ma et al., 2006), but nonlinear computations such as quadratics and divisive normalization are widely implicated in neural processing (Busse et al., 2009; Beck et al., 2011; Carandini and Heeger, 2012; Ni et al., 2012; Louie et al., 2013; Qamar et al., 2013; Rosenberg et al., 2015). These nonlinearities can introduce dependencies between the representations of individual cues, and may therefore limit the precision of perception. Specifically, even if the individual cues were optimally integrated, the perception of combined-cue stimuli would be less precise if their representations were dependent than if they were independent (Oruç et al., 2003). We therefore performed simulations to assess the impact of the neural architecture and computations on perception.

We found that combined-cue perception was consistent with a neural architecture in which stereoscopic cues and perspective cues were represented by independent neuronal populations. To account for perception, a key element was to combine perspective cues detected by the two eyes using quadratic nonlinearities and divisive normalization before their integration with stereoscopic cues. The integration of stereoscopic cues and perspective cues was statistically optimal since the neuronal representations were linearly summed (Ma et al., 2006). However, cue integration was not maximized. An alternative architecture in which stereoscopic cues, left eye perspective cues, and right eye perspective cues were each represented by independent populations yielded two times greater precision. Our findings suggest that cue integration is a conserved computation by which primates achieve robust 3D vision, generate testable hypotheses about neural architectures responsible for 3D perception, and indicate that the concurrence of multiple canonical neural computations (linear summation, quadratics, and divisive normalization) may simultaneously optimize and curb perception.

## Materials and Methods

### Subjects and preparation

All surgeries and experimental procedures were approved by the Institutional Animal Care and Use Committee (IACUC) at the University of Wisconsin–Madison, and in accordance with the National Institutes of Health’s Guide for the Care and Use of Laboratory Animals. Two male rhesus macaques (*Macaca mulatta*) participated (Monkey L: five years of age, ∼7.8 kg in weight; Monkey F: four years, ∼5.5 kg). A Delrin ring for stabilizing the head during training and experimental sessions was attached to the skull under general anesthesia. After recovery, the monkeys were trained to sit in a custom primate chair with head restraint, and to fixate targets for 2 s at simulated depths ranging between –20 and 40 cm from the screen (located at 57 cm) within 2° version and 1° vergence windows for a liquid reward. Eye positions were monitored optically at 1000 Hz (EyeLink 1000 plus, SR Research).

### Experimental control and stimulus presentation

Experimental control was performed using an open-source, network-based parallel processing framework (Kim et al., 2019). Stimuli were created in MATLAB using Psychtoolbox 3 (Kleiner et al., 2007), and rendered with anti-aliasing using an NVIDIA GeForce GTX 970 graphics card on a Linux workstation (Ubuntu 16.04 LTS, Intel Xeon Processor, 24 GB RAM). A DLP LED projector (VPixx Technologies, Inc.) rear projected the stimuli at 1280 × 720-pixel resolution with a 240-Hz refresh rate onto a polarization preserving screen (Stewart Film Screen, Inc.). The projected area subtended 70° × 43° of visual angle. Stereoscopic presentation was achieved by sequencing the presentation of “half-images” to each eye (120 Hz/eye) using a circular polarizer synchronized to the projector. Polarized glasses were worn.

### Visual stimuli

**Figure 2.**
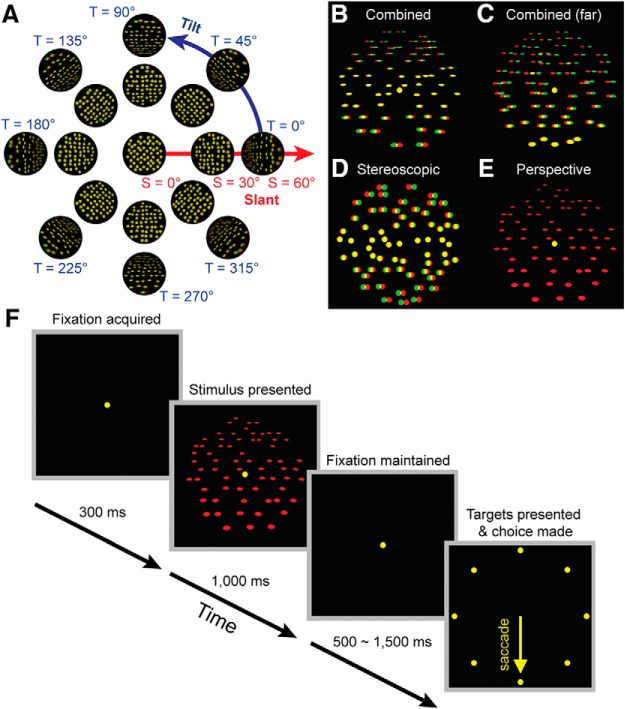
Stimuli and discrimination task. ***A***, Tilt (T) and slant (S) are polar coordinates describing planar surface orientation. Tilt specifies the direction that the plane is oriented in depth. Slant specifies how much it is oriented in depth. ***B–E***, Example planes (T = 270°, S = 60°). For clarity, the dot size is exaggerated and the dot number is reduced from the actual experiments. ***B***, Combined-cue stimulus at 57 cm (fixation distance). ***C***, Combined-cue stimulus at 77 cm (all dots behind the plane of fixation). ***D***, Stereoscopic cue stimulus at 57 cm. ***E***, Perspective cue stimulus at 57 cm (left eye presentation). ***F***, Eight alternative tilt discrimination task. Fixation was held on a target presented at 57 cm (screen distance) for 300 ms. A plane then appeared for 1000 ms. Fixation was then held for 500–1500 ms before the fixation target disappeared and eight choice targets appeared. The plane’s tilt was reported through a saccade to a choice target. For example, the bottom target for a bottom-near plane (T = 270°). Planes are illustrated here using red-green anaglyphs.

Planar surfaces were defined using random dot patterns (*N* = 250 dots). The dots were bright (35.1 cd/m^2^) on a gray (11.06 cd/m^2^) background (PR-524 LiteMate, Photo Research), measured through the glasses. On the screen, the stimulus envelope was circular and subtended 20°. We defined planar surface orientation using two angular variables: tilt and slant (Stevens, 1983b; Rosenberg et al., 2013). Tilt is a rotation about an axis parallel to the line of sight, and therefore specifies the direction that the plane is oriented in depth. Tilt was defined such that: right-near = 0°, top-near = 90°, left-near = 180°, and bottom-near = 270°. Slant is a rotation about an axis perpendicular to the line of sight, and therefore specifies the amount of depth variation. Together, these variables define a polar coordinate system (Fig. 2*A*). Planes were presented at all combinations of eight tilts (0° to 315° in 45° steps), four slants (15° to 60° in 15° steps), and either six (Monkey L: 37, 57, 77, 87, 107, and 137 cm) or eight (Monkey F: 37, 57, 77, 87, 97, 107, 117, and 137 cm) distances. Fixation was always at screen distance (57 cm), regardless of the stimulus distance. The stimulus distances corresponded to horizontal disparity pedestals of: –1.63° (37 cm), 0° (57 cm), 0.78° (77 cm), 1.04° (87 cm), 1.24° (97 cm), 1.41° (107 cm), 1.55° (117 cm), and 1.76° (137 cm). At 37 cm, all dots were in front of the plane of fixation. At 57 cm, dots were distributed in front of and behind the plane of fixation. At 77 cm and beyond, all dots were behind the plane of fixation. The protocol was thus similar to previous studies which varied the stimulus distance while holding the fixation distance constant (Nguyenkim and DeAngelis, 2003; Hegdé and Van Essen, 2005; Ban and Welchman, 2015; Alizadeh et al., 2018; Elmore et al., 2019; Henderson et al., 2019). Others have yoked the stimulus and fixation distances (Banks et al., 2001; Hillis et al., 2004). By presenting stimuli with the dots entirely in front of, distributed about, or entirely behind the plane of fixation, we prevented the monkeys from relying on local absolute disparity cues to perform the discrimination task (described below), ensuring that they judged the plane’s 3D orientation (Elmore et al., 2019).

Three cue conditions were included. For combined-cue stimuli, both stereoscopic and perspective cues signaled the plane’s orientation (Fig. 2*B*,*C*). The dots were uniformly distributed across the plane in the world. Left and right eye half-images were rendered using projective geometry, resulting in retinal density gradients, foreshortening, and scaling. The baseline dot size was 0.35°. For the largest slant tested (60°), the major and minor axes of dots on the axis of rotation were 0.35° × 0.18° (5.6 × 2.8 pixels). At the nearest point, they were 0.45° × 0.29° (7.3 × 4.8 pixels). At the furthest point, they were 0.24° × 0.08° (3.9 × 1.4 pixels). As in previous work (Hillis et al., 2004), the reliability of the perspective cues was held constant with distance by scaling the dots such that their screen size only depended on slant. Stereoscopic cue stimuli were created by defining a uniform distribution of dots on the screen and using ray tracing to assign each dot to a location on the plane in the world (Fig. 2*D*). All dots had a circular shape and subtended 0.35°, irrespective of the plane’s pose. The perspective cue stimuli were the same as the combined-cue stimuli but only one eye saw the plane (pseudo-randomly selected each trial) while both eyes saw the fixation target (Fig. 2*E*).

### Tilt discrimination task

The monkeys were trained to report planar tilt in an 8AFC task. They first learned to perform a right-near versus left-near task with all slants, distances, and cue conditions interleaved. The correct choice target initially had a higher contrast than the distractor, and the contrast difference was reduced with training. Once an 80% correct rate with equal target contrasts was reached for planes at 57 cm, the four cardinal tilts were introduced with a target contrast difference. Once a 50% correct rate was reached with equal contrasts for planes at 57 cm, we started alternating training days between four cardinal and four oblique tilts. Once a 50% correct rate was reached with both sets at 57 cm, all eight tilts were introduced. Data collection began after performance in the 8AFC task stabilized. The correct rate depended on the surface pose and cue condition. Across all stimuli, each monkey had a mean correct rate of 55% and a SD of 27% (Monkey L: *N* = 576; Monkey F: *N* = 768). Both monkeys showed chance-level performance in a relatively small number of stimulus conditions (Monkey L: 45/576, 7.8%; Monkey F: 9/768, 1.2%), but this had no obvious impact on their motivation to perform the task.

In the task (Fig. 2*F*), a monkey first acquired fixation of a target presented at 57 cm in the center of the screen. The target was a red dot (9.6 cd/m^2^ through the glasses) subtending 0.3°. After fixating for 300 ms, a plane was presented at the center of the screen for 1000 ms while fixation was maintained. Fixation was then held for an additional 500–1500 ms (pseudo-random duration) before the fixation target disappeared and 8 choice targets (subtending 0.7°, 35.1 cd/m^2^ through the glasses) appeared at 11° eccentricity and polar angles of 0–315° in 45° steps. The side of the plane nearest the monkey was reported with a saccade to the choice target at the corresponding polar angle for a liquid reward. A trial was aborted if fixation was broken before the choice targets appeared or if a choice was not made within 500 ms, and the data were discarded. In NHP 3D vision studies, vergence is often enforced offline only (e.g., Sanada and DeAngelis, 2014). Similarly, we enforced version and vergence with 2° windows during the task, and offline used a 0.5° window to eliminate trials with vergence errors ≥0.25°.

Stimuli were presented in a pseudo-random order in a block design. A block included one repetition of each combination of tilt, slant, distance, and cue condition (Monkey L: *N* = 576; Monkey F: *N* = 768). Aborted trials were reinserted at pseudo-random locations within the block, which did not finish until a response was provided for each stimulus. The percentage of trials that were aborted did not depend on the tilt, slant, distance, or cue condition for either monkey (χ^2^, all *p* > 0.91). Monkey L completed 16,883 trials. Monkey F completed 52,586 trials.

### Stereoscopic cue controls

We tested whether the stereoscopic cue stimuli contained perspective cues that could be used to perform the task. Stereoscopic cue stimuli were presented binocularly as in the main experiment (both eyes saw the plane) as well as monocularly to eliminate stereoscopic cues (one eye saw the plane, both saw the fixation target) at 57 cm. To maximize potential perspective cues, the planes were presented at the largest tested slant (60°). Parameters were otherwise the same as in the main experiment. All planes were presented interleaved. Monkey L completed 1192 trials. Monkey F completed 1780 trials.

We also tested whether perception of the stereoscopic cue stimuli was affected by a potential conflict between the stereoscopically defined slant of the planes and the isotropic shape of the dots. To maximize the potential conflict, the planes were presented at 60° of slant. Eleven dot numbers ranging from 5 to 250 (steps of 25 starting at 25) were used. The planes were presented at 57 cm for Monkey L, and at 57 and 97 cm for Monkey F. Parameters were otherwise the same as in the main experiment. All planes were presented interleaved. Monkey L completed 3008 trials. Monkey F completed 5521 trials at 57 cm, and 4916 trials at 97 cm.

### Analyses

#### Quantifying tilt perception

To quantify performance, we computed probability density functions describing the errors in reported tilts. First, we took the difference between the reported and presented tilt for each trial: ΔTilt = reported tilt – presented tilt. Second, we built error distributions by calculating the probability of each ΔTilt. The error distributions were calculated using either all tilts or just one tilt, depending on the analysis. Third, a von Mises probability density function was fit to each error distribution:

(1) $$VM\left(\Delta Tilt\right)=e^{\left(\kappa \cdot \text{cos}(\Delta Tilt-\mu )\right)}/(2\pi \cdot I_{0}(\kappa )).$$

The mean (μ) and concentration (*κ*) parameters describe the accuracy and precision of perception, respectively (Murray and Morgenstern, 2010; Seilheimer et al., 2014; Dakin and Rosenberg, 2018). The closer μ is to 0, the more accurate (less biased) the judgments. The larger the *κ*, the more concentrated the distribution, indicating more precise judgments. A modified Bessel function of order 0, *I*_0_(*κ*), normalizes the function to have unit area. The tilt sampling interval limits the maximum *κ* that can be estimated. Simulations showed that we could not distinguish between *κ*'s >18 (≥90% of the density function within the sampling interval), so we set 18 as the upper bound in the estimation routine.

#### Optimal cue integration

We used optimal cue integration theory for circular variables to predict the combined-cue performance (Murray and Morgenstern, 2010). Predictions were derived from the stereoscopic and perspective cue biases (*μ_S_* and *μ_P_*, respectively) and precisions (*κ_S_* and *κ_P_*, respectively). The optimal combined-cue parameters (bias: ${\hat{\mu }}_{C}$; precision: ${\hat{\kappa }}_{C}$) are:

(2) μ^C=tan−1(κS⋅sin(μS)+κP⋅sin(μP)κS⋅cos(μS)+κP⋅cos(μP))

and

(3) $${\hat{\kappa }}_{C}=\sqrt{\kappa_{S}^{2}+\kappa_{P}^{2}+2\cdot \kappa_{S}\cdot \kappa_{P}\cdot \text{cos}(\mu_{S}-\mu_{P}).}$$

#### Estimating a prior over tilt

For each stimulus condition, we used the product of a sensory likelihood function and a prior over tilt to model the perceptual data (Landy et al., 2011):

(4) $$p(T|\hat{T})\propto L(\hat{T}|T)\cdot p(T).$$

The posterior distribution, $p(T|\hat{T})$, indicates the probability that tilt *T* was presented given that tilt $\hat{T}$ was sensed. The likelihood of sensing $\hat{T}$ given *T*, $L(\hat{T}|T)$, was assumed to be an unbiased von Mises function. The prior over tilt, *p*(*T*), was constrained based on the natural scene statistics of surface tilt which includes more cardinal than oblique tilts, and equal left-near and right-near tilts (Adams et al., 2016; Burge et al., 2016). The prior was thus modeled as the mean of four von Mises densities centered on the cardinal tilts (Alberts et al., 2016). The concentration parameters of the densities centered at 0° and 180° were equal to reflect the symmetry of world tilts. Thus, the prior had three free parameters: *κ*_0° & 180°_, *κ*_90°_, and *κ*_270°_.

A prior has the largest effect at low precisions. Since we found little to no bias in tilt perception for most conditions, we estimated the prior using a subset of the data, restricted to the first quartile of *κ*'s (Monkey L: *κ* ≤ 1.59, *N* = 144; Monkey F: *κ* ≤ 1.32, *N* = 192). For this subset of conditions, we fit each likelihood concentration parameter and a common prior by minimizing the root mean square error between the proportion of responses made by the monkeys and simulated with the model by binning 50,000 samples from the posterior into the eight tilt options. We bootstrapped the 95% confidence interval of the prior using the same data (*N* = 1000 repetitions). Concentration parameters for likelihood functions with *κ*'s in the upper three quartiles were then fit using the estimated prior.

For each combination of tilt, slant, and distance, the stereoscopic likelihood function, $L_{S}(\hat{T}|T)$, perspective likelihood function, $L_{P}(\hat{T}|T)$, and common prior were likewise used to predict the combined-cue performance following Bayes’ rule:

(5) p(T|T^)∝LS(T^|T)⋅LP(T^|T)⋅p(T).

For comparison with the behavior, the predicted performance was fit with a von Mises probability density function.

### Neuronal cue integration models

We used Bayesian decoding of modeled 3D orientation-selective neurons to assess how neural computation might constrain tilt perception (Ma et al., 2006; Rosenberg et al., 2015). Assuming independent neurons with Poisson spike count statistics, the probability that tilt *T* elicits population response (***r***) is:

(6) $$p\left(r\text{|}T\right)=\underset{i}{\prod }\frac{e^{-f_{i}\left(T\right)}\cdot f_{i}{\left(T\right)}^{r_{i}}}{r_{i}!}.$$

Here, *f_i_*(*T*) and *r_i_* are the *i* ^th^ neuron’s tilt tuning curve and response, respectively. The posterior, *p*(*T*|***r***), describing the probability that *T* was presented given ***r*** is proportional to Equation 6 (assuming a uniform prior, a simplification we made to focus on computations specific to cue integration and since the influence of a prior was relatively small in our data). As the number of neurons increases, *p*(*T*|***r***) converges to a Gaussian. Assuming *p*(*T*|***r***) guides behavior, the precision of perception (1/*σ* ^2^ for that Gaussian) is proportional to the gain of the population activity (*g*). The proportionality constant (*λ*) depends on the number of neurons and their tuning widths (Ma et al., 2006).

The tuning curves were modeled based on 3D orientation-selective neurons in the caudal intraparietal (CIP) area (Taira et al., 2000; Tsutsui et al., 2002, 2003; Rosenberg et al., 2013). The stimuli used for the neuronal recordings were the same as in the behavioral task, except that only combined-cue stimuli at distances of 37, 57, 97, and 137 cm were shown. The tilt tuning curve at each slant–distance combination was fit with a von Mises function (Rosenberg and Angelaki, 2014a). For each monkey, we calculated the mean response amplitude and tuning width across neurons at each slant–distance combination (Monkey L: *N* = 169; Monkey F: *N* = 180), and linearly interpolated the values for untested distances. These parameters were used to simulate 72 neurons for each monkey, with a 5° spacing between tilt preferences. The model tuning curves were homogeneous in that for a given slant–distance combination, only the preferred tilt differed.

To determine the *λ* relating gain to precision, we minimized the difference between the observed and simulated perceptual precisions decoded after scaling the population responses by *λ*(*κ*_S,D_), which was a function of the slant and distance dependent tuning width (*κ*_S,D_). Four functions were tested: linear, exponential, double exponential, and two-phase exponential. The exponential function, *λ*(*κ*_S,D_) = *DC* + *G* · exp(–*α* · *κ*_S,D_), provided the best fit based on Akaike’s information criterion (Monkey L: *DC* = 1.3 × 10^−3^, *G* = 3.70, *α* = 2.37; Monkey F: *DC* = 1.5 × 10^−3^, *G* = 2.03, *α* = 2.50).

Using these *λ*(*κ*_S,D_) functions, we then fit the response amplitude for each cue-isolated condition to match the monkeys’ precisions. Since CIP tilt tuning widths are similar regardless of the defining cue (Tsutsui et al., 2001, 2002), we assumed that the tuning widths were constant across cue conditions. The tilt tuning curves of the model neurons were:

(7) $$f_{i}\left(T|S,D,C\right)=g_{S,D,C}\,\;\cdot \;{\text{e}}^{\kappa_{S,D}\cdot [\cos\left(T-\mu_{i}\right)-1]}.$$

Here, *f_i_* (*T*|*S*, *D*, *C*) is the *i* ^th^ neuron’s tilt tuning curve for a given slant (*S*), distance (*D*), and cue condition (*C*). The gain (*g_S,D,C_*) depended on the slant, distance, and cue condition. The tuning width (*κ_S,D_*) depended on the slant and distance. The preferred tilt is *μ_i_*.

Stereoscopic (***r****_S_*), left eye perspective ($r_{P_{L}}$), and right eye perspective ($r_{P_{R}}$) population responses were simulated. We tested three architectures for integrating these responses to create a combined-cue representation (**r***_C_*) that was decoded after scaling by *λ*(*κ*_S,D_). Since the tuning curves were homogeneous and the simulations did not incorporate a prior, precision was independent of tilt. All simulations were performed with *T* = 180°. Since Equation 6 converges to a Gaussian, the decoded posteriors were fit with Gaussian probability functions. To allow for direct comparisons, we refit the monkeys’ error distributions with Gaussians. Gaussian and von Mises precision estimates were highly correlated (Monkey L: *r* = 0.99, *p* = 3.8 × 10^−74^; Monkey F: *r* = 0.99, *p* = 1.2 × 10^−80^).

#### Three independent cue-isolated populations

In this architecture, stereoscopic cues, left eye perspective cues, and right eye perspective cues were represented by three independent neuronal populations. Optimal cue integration was performed by a fourth population that linearly summed the three cue-isolated population responses to create a combined-cue representation (Ma et al., 2006), ***r****_C_*:

(8) $$r_{C}=\overset{\text{stereo. pop.}}{\overset{︷}{r_{S}}}+\overset{\text{left eye persp. pop.}}{\overset{︷}{r_{P_{L}}}}+\overset{\text{right eye persp. pop.}}{\overset{︷}{r_{P_{R}}}}.$$

#### Two independent cue-isolated populations

In this architecture, stereoscopic cues and both eyes’ perspective cues were represented by two independent populations. The response of the perspective population to binocular stimulation was the divisively normalized sum of the squared monocular responses. Optimal cue integration was performed by a third population that linearly summed the stereoscopic cue and single perspective cue population responses to create a combined-cue representation:

(9) $$r_{C}=\overset{\text{stereo. pop.}}{\overset{︷}{r_{S}}}+\overset{\text{single persp. pop.}}{\overset{︷}{\frac{r_{P_{L}}{}^{2}+r_{P_{R}}{}^{2}}{r_{P_{L}}+r_{P_{R}}}}}\text{.}$$

#### One population

In this architecture, stereoscopic cues and both eyes’ perspective cues were represented by a single population. The response to binocular stimulation was the divisively normalized sum of the squared cue-isolated responses:

(10) $$r_{C}=\overset{\text{one pop.}}{\overset{︷}{\frac{r_{S}{}^{2}+r_{P_{L}}{}^{2}+r_{P_{R}}{}^{2}}{r_{S}+r_{P_{L}}+r_{P_{R}}}}}\text{.}$$

### Code accessibility

The simulations were performed in MATLAB R2018a on a Windows 10 workstation. The code is available on GitHub: https://github.com/RosenbergLab/Optimized-but-not-maximized-cue-integration-for-3D-visual-perception.

## Results

### The precision of tilt perception depends on slant and distance

Tilt and slant are two angular variables describing 3D planar surface orientation (Stevens, 1983b; Rosenberg et al., 2013). Tilt specifies the direction that the plane is oriented in depth (e.g., right-near = 0°, top-near = 90°), and slant specifies how much it is oriented in depth (Fig. 2*A*). For planar surfaces with different slants, distances, and defining cues (Fig. 2*B–E*), we trained two monkeys to perform an 8AFC tilt discrimination task (Fig. 2*F*).

**Figure 3.**
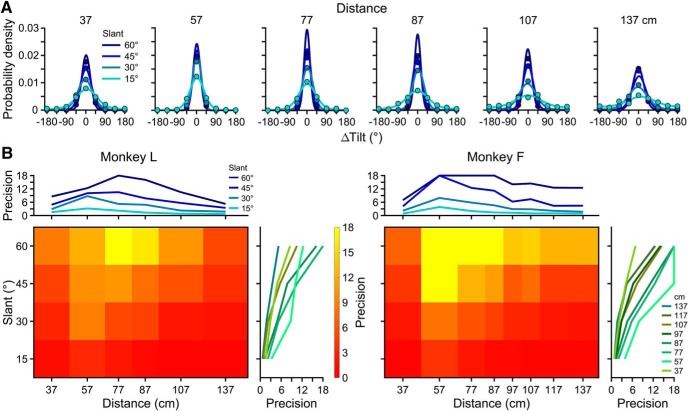
Tilt perception for combined-cue stimuli. ***A***, Probability density functions describing the errors in reported tilts made by Monkey L for each slant–distance combination, calculated using all eight tilts. Columns correspond to distance, and colors to slant. The probability that an error of a given ΔTilt was made is shown with a point. Correct choices: ΔTilt = 0°. Solid curves are fitted von Mises probability density functions. At high precisions, there is some deviation between the point representing the probability that the monkey was correct and the probability density function. This deviation reflects discrete versus continuous representations of the area between sampled tilts, and that the sampling interval limits the maximum *κ* that can be estimated. An upper bound of *κ* = 18 was set based on simulations (see Materials and Methods). ***B***, Heat maps showing the precision (von Mises *κ*) of tilt perception as a function of slant and distance for both monkeys, calculated using all eight tilts. Red hues indicate lower precision and yellow hues indicate higher precision. Right marginals show *κ* as a function of slant for each distance. Precision increased monotonically with slant. Upper marginals show *κ* as a function of distance for each slant. Precision had an inverted U shape as a function of distance. Also see Extended Data Figure 3-1.

We first assessed how the precision of tilt perception depended on slant and distance for combined-cue stimuli defined by stereoscopic and perspective cues. Distributions describing the errors in reported tilts (ΔTilt = reported tilt – presented tilt) were calculated using all 8 presented tilts. Tilt error distributions for each of the 24 slant–distance combinations tested with Monkey L are shown in Figure 3*A*. To quantify performance, we fit von Mises probability density functions (solid curves). A fitted density function’s concentration parameter (*κ*, the circular analog of *σ* ^−2^ for a Gaussian) indicates the precision of perception. Larger *κ* (taller and narrower densities) indicate greater precision. The joint dependency of precision on slant and distance is shown for both monkeys using heat maps in Figure 3*B*.

10.1523/ENEURO.0411-19.2019.f3-1 Extended Data Figure 3-1 Precision and cue integration with larger stimuli. Monkey L also performed the task with 30° planes defined by 550 dots (*N* = 8360 trials). Four slants (15°, 30°, 45°, and 60°) and six distances (37, 57, 77, 97, 137, and 177 cm) were presented. Slant–distance combinations at which performance was not different from chance (Rayleigh test and Bonferroni corrected) are outlined in black. ***A–C***, Heat maps showing the precision (*κ*) of tilt perception as a function of slant and distance, calculated using all eight tilts. ***A***, Combined-cue stimuli. ***B***, Stereoscopic cue stimuli. Performance was at chance level for all slants at 177 cm. ***C***, Perspective cue stimuli. ***D***, Cue integration. Each point shows the optimal versus observed combined-cue precision for a single slant–distance combination (*N* = 24). The Type-II regression line is plotted in yellow. Inset shows the correlation and regression line equation. Download Figure 3-1, TIF file.

**Figure 4.**
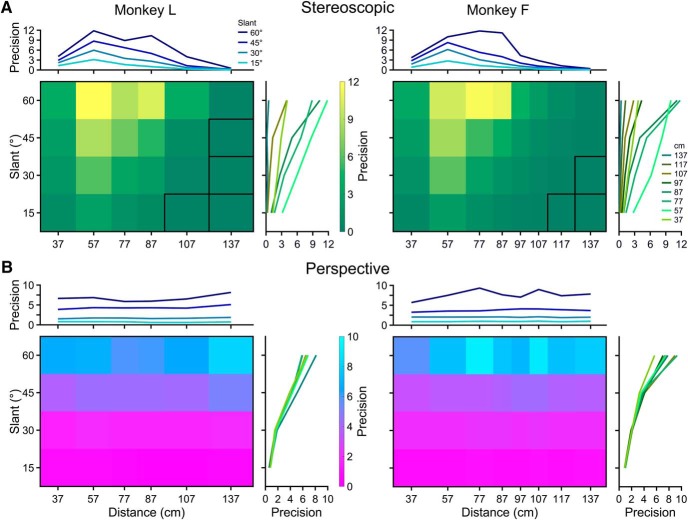
Precision of tilt perception for cue-isolated stimuli. ***A***, Stereoscopic cues. Precision (*κ*) increased monotonically with slant and had an inverted U shape as a function of distance. Performance was at chance level for combinations of small slants and large distances (outlined in black). ***B***, Perspective cues. Precision increased monotonically with slant and was independent of distance. Plots follow the format in Figure 3*B*. Also see Extended Data Figure 3-1.

Combined-cue tilt perception depended on slant and distance in distinct ways. At each distance (Fig. 3*A*, columns), the probability density functions grew taller and narrower with increasing slant. This pattern indicates a monotonic relationship between precision and slant, which is visualized in plots of *κ* versus slant for each distance (Fig. 3*B*, right marginals). The monotonic relationship between precision and slant is consistent with the slant-dependent reliability of perspective cues (Fig. 1*B*). Precision had an inverted U shape as a function of distance. This pattern can be seen by comparing probability density functions across columns in Figure 3*A*. For each slant, the density functions were most concentrated at or just behind the plane of fixation (57 cm). The inverted U-shape relationship between precision and distance is visualized in plots of *κ* versus distance for each slant (Fig. 3*B*, top marginals). Decreasing precision with distance is consistent with the distance-dependent reliability of stereoscopic cues (Fig. 1*A*). The falloff in precision with distance from the fixation plane (both toward and away from the monkey) is consistent with the limited range of horizontal disparities represented by the visual system (Prince et al., 2002). For example, this explains why greater falloffs in precision occurred between 57 and 37 cm than between 57 and 77 cm, since the magnitude of the disparity pedestal at 37 cm (–1.63°) was bigger than at 77 cm (0.78°). Asymmetric falloffs were especially evident in the stereoscopic cue data, described in the next section (Fig. 4*A*).

The interaction of the monotonic relationship between precision and slant and the inverted U-shape relationship between precision and distance resulted in a more gradual falloff in precision with distance at larger slants, giving the heat maps a wedge-shaped appearance (Fig. 3*B*). From these plots, it is apparent that precision peaked at the largest tested slant (60°), and either at or just behind the plane of fixation. The mean precision across all slant–distance combinations was *κ* = 6.19 ± 4.81 SD for Monkey L (*N* = 24), and 7.03 ± 5.92 for Monkey F (*N* = 32). Although the precision of tilt perception varied substantially as a function of slant and distance, performance was always above chance (Rayleigh test for circular uniformity, all *p* ≤ 2.3 × 10^−7^, Bonferroni corrected for 24 or 32 comparisons for Monkey L or F, respectively). A similar pattern was found with larger stimuli over a wider range of distances (Extended Data Fig. 3-1*A*). These results suggest that the precision of tilt perception depended on a combination of physiological factors and the pose-dependent reliabilities of the visual cues.

### Contributions of stereoscopic cues to tilt perception

We next assessed the contributions of stereoscopic cues to tilt perception by analyzing responses to planar surfaces which had no perspective cues that could be used to perform the task (Fig. 2*D*). Error distributions of reported tilts were calculated using all eight tilts, and fit with von Mises densities to quantify performance. The precision of tilt perception based on stereoscopic cues is plotted as a function of slant and distance in Figure 4*A*.

The mean precision across all slant–distance combinations was *κ* = 3.63 ± 3.45 SD for Monkey L (*N* = 24), and 2.87 ± 3.29 for Monkey F (*N* = 32). We found a monotonic relationship between precision and slant, indicating that the monkeys were able to more reliably estimate the direction of the gradient of horizontal disparities at larger slants. There was also an inverted U-shape relationship between precision and distance which, as discussed above, was consistent with a combination of geometric and physiological factors.

The stereoscopic cue and combined-cue precision landscapes were similar in shape (Monkey L: *r* = 0.89, *p* = 4.4 × 10^−9^; Monkey F: *r* = 0.82, *p* = 1.1 × 10^−8^). However, precision was significantly lower with stereoscopic cue than combined-cue stimuli (Wilcoxon signed-rank test; Monkey L: *p* = 1.8 × 10^−5^, *N* = 24; Monkey F: *p* = 8.0 × 10^−7^, *N* = 32). At combinations of small slants and large distances, performance with the stereoscopic cue stimuli was not significantly different from chance (Fig. 4*A*, conditions outlined in black; Rayleigh test and Bonferroni corrected). At greater distances, performance was at chance level even with larger stimuli (Extended Data Fig. 3-1*B*). These findings indicate that stereoscopic cues did not contribute to tilt perception beyond ∼137 cm, and imply that perspective cues were entirely responsible for above chance performance with combined-cue stimuli at some poses.

**Figure 5.**
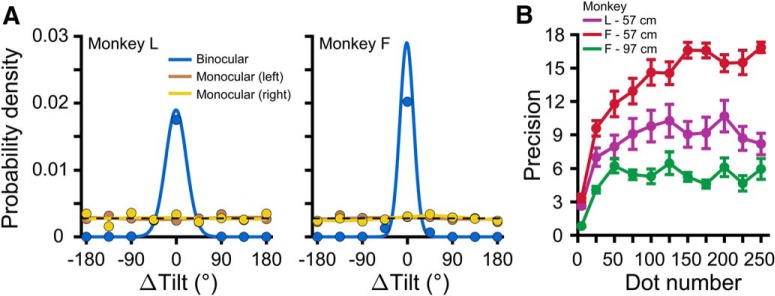
Stereoscopic cue controls. ***A***, Stereoscopic cue stimuli were viewed binocularly (blue curves) or monocularly (left eye stimulated: orange; right eye stimulated: yellow). Probability density functions describing the errors in reported tilts made by each monkey, calculated using all eight tilts are plotted. The probability that an error of a given ΔTilt was made is shown with a point. Correct choices: ΔTilt = 0°. Solid curves are fitted von Mises probability density functions. Chance performance is indicated by dashed black lines. ***B***, Precision (*κ*) versus dot number for planes at 57 cm (Monkey L: purple; Monkey F: red) and 97 cm (Monkey F: green). Error bars are SEM across sessions.

We performed a control experiment to verify that the stereoscopic cue stimuli had no perspective cues that could be used to perform the task (see Materials and Methods). Stereoscopic cue stimuli were presented binocularly (both eyes saw the plane) as well as monocularly (one eye saw the plane). Above chance performance with monocular viewing would indicate that there were usable perspective cues. As expected, the monkeys performed well with binocular viewing (Rayleigh test; Monkey L: *p* = 2.2 × 10^−308^; Monkey F: *p* = 2.2 × 10^−308^). In contrast, monocular performance was not significantly different from chance (Monkey L: left eye *p* = 0.79, right eye *p* = 0.91; Monkey F: left eye *p* = 0.77, right eye *p* = 0.11). Thus, the stereoscopic cue stimuli contained no usable perspective information for performing the task (Fig. 5*A*).

A second control examined whether performance with the stereoscopic cue stimuli was affected by a potential conflict between the stereoscopically defined planar slant (which was always non-zero) and the isotropic shape of the dots which could be interpreted as signaling zero slant. This conflict would increase with greater dot number, causing a decrease in precision (Hillis et al., 2004). We therefore assessed how the precision of stereoscopic cue perception depended on dot number. As expected, precision depended significantly on dot number (Kruskal–Wallis test; Monkey L: *p* = 2.7 × 10^−5^; Monkey F: *p* = 9.3 × 10^−13^ at 57 cm; Monkey F: *p* = 1.7 × 10^−9^ at 97 cm). Precision initially increased, reflecting that more dots provide greater signal for performing the task. To test for decreases in precision, we ran pairwise comparisons using Tukey’s HSD test. In each case of a significant difference, the precision at the larger dot number was greater than at the smaller number. There were no significant differences between precisions at dot numbers ≥75. These results suggest that precision increased monotonically with dot number, and thus that performance was not affected by this potential conflict (Fig. 5*B*).

### Contributions of perspective cues to tilt perception

We next assessed the contributions of perspective cues to tilt perception by analyzing responses to planes lacking stereoscopic cues (Fig. 2*E*). Stereoscopic cues were eliminated by presenting combined-cue stimuli to one eye only. Left and right eye projections of 3D stimuli are not copies of each other, and sensitivity can differ between the eyes, at least for perspective-defined 3D motion (Thompson et al., 2019). We therefore tested whether the precision of tilt perception depended on which eye saw the plane. Error distributions of reported tilts were calculated separately for each eye using all eight tilts, and fit with von Mises densities to compare performance. Precision did not significantly depend on which eye was stimulated (Wilcoxon signed-rank test; Monkey L: *p* = 0.80; Monkey F: *p* = 0.08), so responses to left and right eye presentations were pooled and fit with von Mises densities to quantify performance. The precision of tilt perception based on perspective cues is plotted as a function of slant and distance in Figure 4*B*.

The mean precision across all slant–distance combinations was *κ* = 3.34 ± 2.42 SD for Monkey L (*N* = 24), and 3.59 ± 2.66 for Monkey F (*N* = 32). Performance was significantly above chance at all poses (Rayleigh test, all *p* ≤ 1.5 × 10^−12^, Bonferroni corrected). Consistent with the slant-dependent reliability of perspective cues (Fig. 1*B*), precision increased monotonically with slant. Reflecting that the perspective cues only signaled 3D orientation (see Materials and Methods), precision was independent of distance. A similar pattern was found with larger stimuli over a wider range of distances (Extended Data Fig. 3-1*C*).

Precision was significantly lower with perspective cue than combined-cue stimuli (Wilcoxon signed-rank test; Monkey L: *p* = 4.9 × 10^−4^; Monkey F: *p* = 2.9 × 10^−6^). The perspective cue and stereoscopic cue precisions were not significantly different when compared across all slants and distances (Wilcoxon signed-rank test; Monkey L: *p* = 0.53; Monkey F: *p* = 0.13). However, the relative precisions for the two cues were distance dependent. For distances at or just behind the plane of fixation (57, 77, and 87 cm), precision was significantly higher with stereoscopic cues (Wilcoxon signed-rank test; Monkey L: *p* = 4.9 × 10^−4^, *N* = 12; Monkey F: *p* = 3.4 × 10^−3^, *N* = 12). For nearer (37 cm) and further distances (>87 cm), precision was significantly higher with perspective cues (Wilcoxon signed-rank test; Monkey L: *p* = 4.9 × 10^−3^, *N* = 12; Monkey F: *p* = 8.9 × 10^−5^, *N* = 20). These findings demonstrate that both stereoscopic cues and perspective cues contribute to 3D perception within peripersonal space, and that perspective cues extend perception beyond the range supported by stereopsis.

### A 3D analog of the oblique effect and priors over surface tilt

Humans perceive 2D tilt more accurately and precisely at cardinal than oblique tilts (the “oblique effect”; Campbell et al., 1966; Westheimer and Beard, 1998). Similar findings were recently reported for human 3D tilt perception (Kim and Burge, 2018). To test whether the monkeys showed a 3D analog of the oblique effect, we fit the error distribution of reported tilts for each combination of tilt, slant, distance, and cue condition with a von Mises density function. We then compared the accuracy and precision of perception across all slant–distance combinations as a function of tilt. Analyses were performed separately for each cue condition.

#### Accuracy results

**Figure 6.**
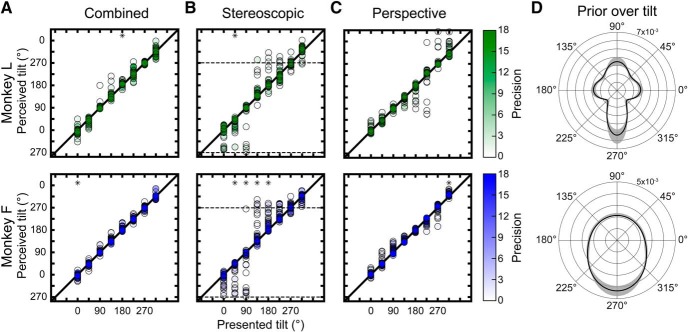
Biases in tilt perception occurred at low precisions and were consistent with a prior over 3D tilt. ***A–C***, Perceived tilt (presented tilt + mean of the von Mises fit to the error distribution) versus presented tilt (Monkey L: *N* = 24 slant–distance combinations per tilt; Monkey F: *N* = 32 per tilt). Diagonals are identity lines. Greater vertical distance from the identity line indicates greater bias. The fill opacity indicates precision (*κ*). Asterisks mark biases that were significantly different from 0°. ***A***, Combined-cue stimuli. ***B***, Stereoscopic cue stimuli. At low precisions, perception was pulled toward 270° (bottom-near), marked by horizontal dashed lines. ***C***, Perspective cue stimuli. ***D***, Priors over 3D tilt. The angular variable is surface tilt and the radial variable is the probability density value. Shading indicates the bootstrapped 95% confidence interval.

For combined-cue stimuli, the biases at any given tilt were generally small and not significant (Fig. 6*A*). Across the two monkeys, biases that were much smaller than the tilt sampling interval but statistically significant were found at two tilts (one-sample circular median test, *p* < 0.05 and Bonferroni corrected for eight tilts). Monkey L had a significant bias at 180° (median μ = 5.31°, *N* = 24 slant–distance combinations). Monkey F had a significant bias at 0° (median μ = –3.73°, *N* = 32). Both biases were in the direction of 270° (bottom-near). We also compared biases between cardinal and oblique tilts. For Monkey L, the median absolute bias at cardinal tilts was 3.81° (*N* = 24 slant–distances × 4 tilts = 96), and at oblique tilts was 8.79° (*N* = 96). Consistent with an oblique effect, the cardinal biases were significantly smaller than the oblique biases (two-sample circular median test, *p* = 1.5 × 10^−3^). For Monkey F, the median absolute biases at cardinal (3.30°, *N* = 128) and oblique (2.89°, *N* = 128) tilts were not significantly different (*p* = 0.62).

For stereoscopic-cue stimuli, the biases at any given tilt were typically small and not significant, but some large biases were evident (Fig. 6*B*). Across the two monkeys, significant biases were found at five tilts. As indicated by the opacity of the data points, large biases were restricted to low precisions (which occurred at small slants and large distances; Fig. 4*A*). Thus, perception was accurate so long as sufficient information was available to perform the task well. Between all cardinal and all oblique tilts, Monkey L’s biases were consistent with an oblique effect: the median absolute bias at cardinal tilts (7.81°) was significantly smaller (two-sample circular median test, *p* = 1.5 × 10^−3^) than at oblique tilts (15.58°). For Monkey F, the median absolute biases at cardinal (9.36°) and oblique (8.31°) tilts were not significantly different (*p* = 0.62). However, when precision was low, the responses of both monkeys were biased toward 270°.

For perspective cue stimuli, the biases at any given tilt were generally small and not significant (Fig. 6*C*). The only significant bias was for Monkey F at 315° (median μ = 9.28). Between all cardinal and all oblique tilts, Monkey L’s biases were consistent with an oblique effect: the median absolute bias at cardinal tilts (5.5°) was significantly smaller (two-sample circular median test, *p* = 1.8 × 10^−4^) than at oblique tilts (8.79°). For Monkey F, the median absolute biases at cardinal (5.67°) and oblique (6.67°) tilts were not significantly different (*p* = 0.45).

#### Precision results

For the combined-cue stimuli, precision was not significantly dependent on tilt for either monkey (Kruskal–Wallis test; Monkey L: *p* = 0.09; Monkey F: *p* = 0.89). After grouping precisions into cardinal and oblique tilts, Monkey L showed an oblique effect: the median precision at cardinal tilts (7.38, *N* = 96) was significantly larger (Mann–Whitney *U* test, *p* = 0.01) than at oblique tilts (5.28). For Monkey F, the median precisions at cardinal (5.12, *N* = 128) and oblique (5.07, *N* = 128) tilts were not significantly different (*p* = 0.79).

For the stereoscopic cue stimuli, precision was not significantly dependent on tilt for either monkey (Kruskal–Wallis test; Monkey L: *p* = 0.70; Monkey F: *p* = 0.21). Precisions were not significantly different between cardinal (Monkey L: median *κ* = 3.34; Monkey F: median *κ* = 1.82) and oblique (Monkey L: median *κ* = 3.04; Monkey F: median *κ* = 1.77) tilts for either monkey (Mann–Whitney *U* test; Monkey L: *p* = 0.36; Monkey F: *p* = 0.98).

For the perspective cue stimuli, we found some tilt-dependent differences. For Monkey L, precision was significantly greater at 90° (median *κ* = 5.70) than 225° (median *κ* = 1.64; Kruskal–Wallis test followed by Tukey’s HSD test, *p* = 1.9 × 10^−3^) or 315° (median *κ* = 2.53; *p* = 4.7 × 10^−2^). For Monkey F, precision was significantly greater at 135° (median *κ* = 4.69; *p* = 4.7 × 10^−2^) than 45° (median *κ* = 2.07). Likewise, precisions were significantly greater at 135° (*p* = 8.5 × 10^−4^) and 225° (median *κ* = 3.59; *p* = 0.02) than at 270° (median *κ* = 1.83). After grouping precisions into cardinal and oblique tilts, Monkey L showed an oblique effect: the median precision at cardinal tilts (4.28) was significantly larger (Mann–Whitney *U* test, *p* = 0.02) than at oblique tilts (3.22). For Monkey F, the median precisions at cardinal (2.74) and oblique (3.08) tilts were not significantly different (*p* = 0.11).

These analyses revealed a 3D analog of the oblique effect in Monkey L, but not Monkey F. Individual differences in the 2D oblique effect are also observed in humans (Westheimer and Beard, 1998; Girshick et al., 2011). However, even in Monkey L, the effects were not prominent since: (1) biases were typically small, (2) precision results varied across cue conditions, and (3) differences between cardinal and oblique tilts required pooling to detect. Nonetheless, both monkeys’ biases appeared consistent with the influence of a prior resembling the 3D tilt statistics of natural scenes (Adams et al., 2016; Burge et al., 2016).

#### Priors over surface tilt

Consistent with the effect of a prior, biases were most apparent at low precisions (Fig. 6*A–C*) and absolute bias was negatively correlated with precision: Spearman *r* = –0.64, *p* = 1.9 × 10^−157^ (*N* = 1344 tilt × slant × distance × cue condition, both monkeys). At the lowest precisions (stereoscopic cue stimuli with small slants and large distances), both monkeys were biased toward 270°. Monkey L, but not Monkey F, also had larger biases at oblique than cardinal tilts. This monkey-specific difference may reflect different internalizations of a prior over tilt. To estimate the prior that best accounted for each monkey’s biases, the perceptual data were modeled using the product of sensory likelihood functions and a prior over tilt (see Materials and Methods). The model described the data well. The circular correlation between observed and predicted perceived tilts was *r* = 0.92, *p* = 2.2 × 10^−308^ (*N* = 1344). Likewise, the Pearson correlation between observed and predicted precisions was *r* = 0.94, *p* = 2.2 × 10^−308^.

The estimated priors captured the monkey-specific biases. Monkey L’s prior had left/right-near lobes that were smaller than the top-near lobe, and a global peak at the bottom-near lobe (Fig. 6*D*, top; fitted prior *κ*'s: *κ*_0° & 180°_ = 2.75, *κ*_90°_ = 3.5, and *κ*_270°_ = 8.5). Net pull due to the lobes’ asymmetries accounts for larger oblique than cardinal biases. Monkey F did not show an oblique effect, and correspondingly the prior did not have four distinct cardinal lobes (Fig. 6*D*, bottom; *κ*_0° & 180°_ = 0, *κ*_90°_ = 0, and *κ*_270°_ = 2). The global peaks at 270° account for the bottom-near biases observed for both monkeys at the lowest precisions. Individual differences in biases were thus consistent with different internalizations of the natural scene statistics of surface tilt, which include more cardinal than oblique tilts and a preponderance of ground planes (Adams et al., 2016; Burge et al., 2016).

### Perceptual cue integration

Perception was more precise with combined-cue than cue-isolated stimuli, and the relative cue-isolated precisions were pose dependent. Given these results, we tested whether the performance with combined-cue stimuli was consistent with an optimal integration strategy. Optimal predictions were derived using the cue-isolated data (see Materials and Methods). We then compared the predictions to the observed combined-cue data to assess whether stereoscopic cues and perspective cues (pooled over the left and right eyes) were dynamically reweighted on a trial-by-trial basis according to their pose-dependent reliabilities.

**Figure 7.**
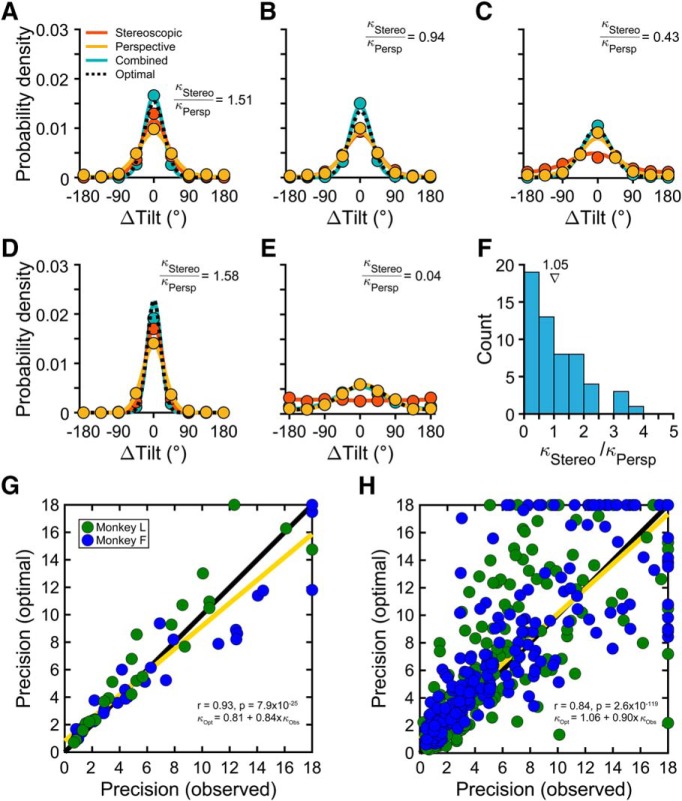
Perceptual cue integration. ***A–E***, Example densities for each cue condition, calculated using all eight tilts. Solid curves are von Mises fits. Dotted black curves are optimal predictions. Insets show cue-isolated *κ* ratios. ***A***, Slant = 30°, distance = 77 cm (Monkey F). ***B***, Slant = 30°, distance = 87 cm (Monkey F). ***C***, Slant = 30°, distance = 107 cm (Monkey L). ***D***, Slant = 45°, distance = 77 cm (Monkey L). ***E***, Slant = 15°, distance = 137 cm (Monkey F). Combined-cue perception depended entirely on perspective cues. ***F***, Distribution of cue-isolated *κ* ratios (*N* = 56 slant–distance combinations, both monkeys). The triangle marks the mean ratio. ***G***, Optimal versus observed combined-cue precision calculated using all eight tilts for each slant–distance combination (*N* = 56). ***H***, Optimal versus observed combined-cue precision calculated for each tilt × slant × distance combination (*N* = 448). Type-II regression lines are shown in yellow (*κ* = 18 excluded). Insets show correlations and regression line equations. Also see Extended Data Figure 3-1.

Example error distributions calculated using all eight tilts are shown for the cue-isolated and combined-cue data (from Figs. 3, 4) along with optimal predictions (derived using Eqs. 2, 3) in Figure 7*A–E*. The observed combined-cue performance (blue curves) was well described by the model (dotted black curves) over a wide range of precisions. The predictions were strong even if only one cue contributed to perception (Fig. 7*E*). Across all slant–distance combinations (*N* = 56, both monkeys), the mean stereoscopic/perspective *κ* ratio was 1.05, which is ideal for evaluating cue integration (Fig. 7*F*). At the same time, the spread of relative cue-isolated precisions allowed us to assess cue integration over a range of naturally occurring cue reliability conditions. The observed and optimal combined-cue precisions were well correlated (*r* = 0.93, *p* = 7.9 × 10^−25^) and distributed along the identity line (Fig. 7*G*), indicating that performance was consistent with an optimal integration strategy. Similar results were found with larger stimuli over a wider range of distances (Extended Data Fig. 3-1*D*).

We performed a second test of cue integration using the likelihood functions and priors estimated in the previous section. For each combination of tilt, slant, and distance (data in Fig. 6; *N* = 448, both monkeys), an optimal prediction of the combined-cue performance was made using the product of the cue-isolated likelihood functions and prior over tilt (Eqs. 4, 5). The observed and optimal perceived tilts were well correlated (circular *r* = 0.98, *p* = 2.2 × 10^−308^), as were the *κ*'s (Pearson *r* = 0.84, *p* = 2.6 × 10^−119^; Fig. 7*H*). The greater scatter between observed and optimal *κ*'s when cue integration was assessed at each tilt (Fig. 7*H*) compared to assessments based on all eight tilts (Fig. 7*G*) was not surprising since the amount of data in the two analyses differed by ∼8-fold. Furthermore, only the second test of cue integration included a prior over tilt. Finding that both optimal precision estimates matched the data well is another indication that the prior had an overall small effect on perception. Together, these findings are consistent with robust 3D perception being achieved through the optimal integration of stereoscopic cues and perspective cues (pooled over the left and right eyes). Since the stimuli were interleaved pseudo-randomly, cue reweighting must have occurred dynamically to match the vagaries of cue reliabilities that occurred with the trial-to-trial changes in surface pose.

### Neuronal models of 3D visual cue integration

We found that combined-cue tilt perception was consistent with the optimal integration of stereoscopic cues and perspective cues (pooled over the left and right eyes). Similar conclusions can be drawn from human slant perception studies (Knill and Saunders, 2003; Hillis et al., 2004). These findings are somewhat surprising since there are actually three cues available for making 3D inferences: stereoscopic cues, left eye perspective cues, and right eye perspective cues. If all three cues were represented independently and optimally integrated, perception would be more precise than observed (Oruç et al., 2003). This observation motivated us to test how the neural architecture and computations responsible for cue integration might constrain the precision of combined-cue tilt perception. By comparing the perceptual data to simulation results, we generate predictions about where and how neural computation might curb 3D perception.

**Figure 8.**
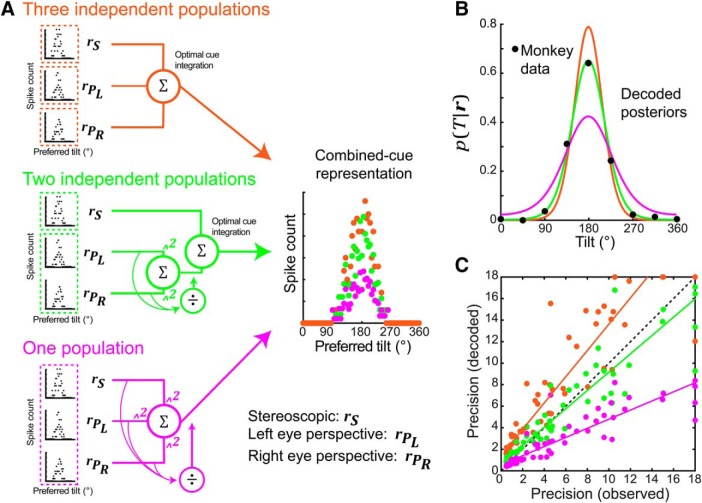
Optimized but not maximized cue integration. ***A***, Schematics of three architectures for combining responses to stereoscopic cues (***r****_S_*), left eye perspective cues ($r_{P_{L}}$), and right eye perspective cues ($r_{P_{R}}$). Top, Three independent populations represent each cue (orange). Middle, Two independent populations represent stereoscopic cues and perspective cues from both eyes (green). Bottom, One population represents all cues (magenta). Right, Combined-cue representations for each architecture. Points show the response of each model neuron, ordered along the *x*-axis by preferred tilt, to a single stimulus presentation (slant = 30°, distance = 37 cm). ***B***, Tilt posteriors, *p*(*T*|***r***), decoded from the combined-cue representations. Black dots show corresponding data from Monkey L. Given the same cue-isolated responses, precision was greatest for the three independent populations model and lowest for the one population model. The posterior of the two independent populations model matched the monkey’s data. ***C***, Comparisons of decoded model precisions and observed monkey precisions. Each point corresponds to one slant–distance combination (*N* = 56, both monkeys). The three independent populations model was more precise than the monkeys (nearly all points are above the dashed black identity line). The precisions from the two independent populations model matched the monkeys’ precisions (points are distributed along the identity line). The one population model was less precise than the monkeys (nearly all points are below the identity line). Solid lines are Type-II regressions (*κ* = 18 excluded).

Performance on the task was simulated using Bayesian decoding of modeled neuronal population responses. Responses to stereoscopic cues, left eye perspective cues, and right eye perspective cues were simulated based on CIP area neurons (see Materials and Methods). Neurons in CIP are selective for 3D surface orientation (Taira et al., 2000; Rosenberg et al., 2013), and respond to both stereoscopic and perspective cues (Tsutsui et al., 2001; Rosenberg and Angelaki, 2014b). Furthermore, their inactivation impairs 3D perception (Tsutsui et al., 2001; Van Dromme et al., 2016), and their activity correlates with surface orientation reports (Elmore et al., 2019). We tested three architectures for combining cue-isolated responses to create a combined-cue representation (Fig. 8*A*). To focus on computations underlying cue integration, and since the effects of a prior were relatively small, we simplified the model by not incorporating a prior over tilt. Thus, perceptual biases were not modeled. For each architecture, we decoded the combined-cue representation to simulate perceptual data (Fig. 8*B*). Decoded model precisions were compared to the monkey precisions (estimated using all eight tilts; Fig. 3 data) for each slant–distance combination (Fig. 8*C*).

The first architecture assumed that each of the three cues was represented by an independent neuronal population. A fourth population linearly summed the three cue-isolated population responses to perform optimal cue integration (Ma et al., 2006), thereby creating a combined-cue representation (Fig. 8*A*, top). This architecture sets an upper bound on combined-cue precision given the cue-isolated precisions. As anticipated, since the perceptual data were consistent with the integration of stereoscopic cues and pooled left and right eye perspective cues, this architecture produced significantly greater precisions than shown by the monkeys: Wilcoxon signed-rank test, *p* = 5.2 × 10^−9^ (Fig. 8*C*, orange points). Importantly, this simulation showed that if the neural network responsible for tilt perception represented the three cues independently and integrated them optimally, then the precision of combined-cue perception would be 1.97 times greater than observed (average decoded/observed precision ratio). Although tilt perception was less precise than theoretically possible, this result does not imply that perception was suboptimal since the monkeys’ performance could reflect the optimal integration of non-independent neuronal representations (Oruç et al., 2003).

The second architecture explored this possibility. Given the perceptual data, stereoscopic cues and both eyes’ perspective cues were assumed to be represented by two independent populations and optimally integrated. Thus, the critical factor was how the perspective cue population combined the response components contributed from each eye, since this determines the level of dependency between them. We found that it was possible to account for the perceptual data if the perspective cue population combined the left and right eye response components using quadratic nonlinearities and divisive normalization (Fig. 8*A*, middle). A similar model describes V1 responses to compound stimuli, and the combination operations are widely implicated in neural processing (Busse et al., 2009; Beck et al., 2011; Carandini and Heeger, 2012; Ni et al., 2012; Louie et al., 2013; Rosenberg et al., 2015). Importantly, these computations made the left and right eye perspective cue representations fully dependent, precluding any improvement in perceptual precision that could have resulted from having two perspective cues. Nevertheless, the stereoscopic cue and perspective cue representations were optimally integrated since the two cue-isolated population responses were linearly summed to create a combined-cue representation (Ma et al., 2006). This architecture produced precisions that were not significantly different from the monkeys’ precisions: Wilcoxon signed-rank test, *p* = 0.78 (Fig. 8*C*, green points). This result suggests that the combination of left and right eye perspective cues using nonlinear canonical computations may be a key factor limiting the precision of tilt perception.

We lastly considered a single neuronal population that estimated tilt from stereoscopic cues as well as both eyes’ perspective cues. For consistency with the two independent populations model, response components driven by each of the three cues were combined with quadratic nonlinearities and divisively normalized (Fig. 8*A*, bottom). Thus, none of the cues were represented independently and no integration of cue-isolated population responses was required to create a combined-cue representation. As anticipated based on the perceptual data and the results from the two independent populations model, this architecture produced significantly lower precisions than shown by the monkeys: Wilcoxon signed-rank test, *p* = 2.5 × 10^−9^ (Fig. 8*C*, magenta points).

These simulations revealed a neural architecture and computations that could account for the 3D perceptual cue integration results. In particular, they pinpointed the processing of left and right eye perspective cues by a single neuronal population as a factor that could substantially limit the precision of tilt perception. They further suggest that tilt perception is consistent with an optimal integration strategy but not maximized due to the concurrence of several canonical computations.

## Discussion

We evaluated the contributions of stereoscopic cues and perspective cues to tilt perception in macaque monkeys. Tilt discrimination was used to study how 3D perception depends on surface pose (position and orientation) since stereopsis and projective geometry dictate that the reliability of these cues depends on distance and slant (Fig. 1). To date, few studies have investigated tilt perception in humans (Stevens, 1983a; Kim and Burge, 2018) or monkeys (Tsutsui et al., 2001, 2003). We found that tilt perception was accurate across a wide range of poses. Instances of poor accuracy were associated with low precision, and presumably reflected low certainty. Precision was highly pose dependent. With stereoscopic cues, precision increased with slant and decreased with distance from the fixation plane. With perspective cues, precision increased with slant. Perspective cues were the sole contributor to tilt perception at small slant and large distance combinations, indicating that they extend 3D perception beyond the range supported by stereopsis. Notably, these results characterize performance on a task that can be used to study the impact of nuisance variables (slant, distance, and visual cues) on neural coding during abstracted sensory (tilt) discriminations.

### Natural scene statistics may influence 3D perception

The 2D oblique effect is characterized by smaller biases and larger precisions at cardinal than oblique tilts (Campbell et al., 1966; Westheimer and Beard, 1998; Girshick et al., 2011). It is thought to be associated with a prior over orientation that reflects natural scene statistics (Coppola et al., 1998). An oblique effect for 3D tilt was recently found in humans (Kim and Burge, 2018). We assessed whether the monkeys showed a 3D oblique effect, while testing for individual differences and cue-specific dependencies. Monkey L had smaller biases at cardinal than oblique tilts in all three cue conditions, and higher precisions at cardinal than oblique tilts in the combined-cue and perspective cue conditions. These tilt dependencies were not found in Monkey F. This suggests that, like the 2D oblique effect (Westheimer and Beard, 1998; Girshick et al., 2011), the 3D oblique effect is subject to individual differences. Given the extensive training with cardinal and oblique tilts, it is unlikely that training induced the oblique effect in Monkey L.

Both monkeys’ reports were biased toward 270° (bottom-near) when precision was especially low. Correspondingly, the estimated priors for both monkeys had global peaks at 270°. This finding is consistent with the influence of a prior for ground planes, which are prominent in natural scenes (Adams et al., 2016; Burge et al., 2016). It is unlikely that a bottom-near bias reflected a preference for making downward saccades since horizontal eye movements are more accurate than vertical eye movements in humans (Kapoula et al., 2010; Ke et al., 2013), and the oculomotor systems of macaques and humans are similar (Fuchs, 1967). The estimated priors also captured the monkey-specific biases. In particular, only Monkey L’s prior had four prominent but asymmetric cardinal lobes, accounting for larger oblique than cardinal biases. These findings are consistent with perception being influenced by a prior resembling the 3D tilt statistics of natural scenes, and further suggest that the internalization of the prior differs across individuals.

### Potential conflicts in stereoscopic cue stimuli

For stereoscopic cue stimuli, there is a potential conflict at non-zero slants since isotropic dots are consistent with zero slant. Supporting this possibility, a previous study found that the precision of slant perception based on stereoscopic cues decreased as the stimulus dot number increased beyond 64 for one observer (Hillis et al., 2004). However, we did not find this result. What may account for the difference? One possibility is that the previously observed decrease in precision may have been unrelated to the potential conflict. The same participant showed a similar pattern with stimuli whose perspective cues signaled the stereoscopically defined reference slant, thus eliminating the relevant conflict. Consistent with this possibility, we found that if performance with stereoscopic cue stimuli was at chance level, performance with combined-cue stimuli was no better than with perspective cue stimuli (Fig. 7*E*). Had a conflict caused the stereoscopic cue precision to be underestimated, the combined-cue precision would have exceeded the perspective cue precision. There may also be individual differences in the perception of such conflicts, perhaps reflecting differences in the strength of a prior for isotropic visual elements (Knill, 1998b). Our results suggest that, at least in monkeys, an isotropy prior is weak or non-existent. Cognitive processes could also be at play if observers reason that circular dots imply zero slant. Future work can evaluate the extent to which such conflicts (when/if they occur) reflect true perceptual conflicts or cognitive reasoning (likely task, training, and instruction dependent).

### Optimal cue integration

We found that the monkeys achieved robust 3D perception by integrating distinct visual cues. Specifically, the precision of tilt perception was consistent with the optimal integration of independently represented stereoscopic cues and perspective cues (left and right eyes pooled). This result is consistent with previous human slant perception findings (Knill and Saunders, 2003; Hillis et al., 2004). Given that fully independent representations are unlikely (e.g., due to common retinal processing), this body of work suggests that the major sources of noise in 3D orientation estimation based on stereoscopic cues and perspective cues are largely independent. Otherwise, the precision of combined-cue perception would have been worse than observed due to correlated errors, even if the cues were optimally integrated (Oruç et al., 2003). Perception could also have been more precise than observed since there were actually three cues that could contribute to 3D orientation estimates in these studies: stereoscopic cues, left eye perspective cues, and right eye perspective cues. One explanation for the level of precision observed is that two distinct neuronal populations create 3D orientation estimates: one using stereoscopic cues and one using perspective cues from both eyes (with left and right eye signals combined in a way that makes them fully dependent). Our simulations, discussed in the next section, explored how this might occur.

Since we interleaved the stimuli pseudo-randomly, our findings imply that stereoscopic cues and perspective cues are dynamically reweighted to match the vagaries of cue reliabilities that occur with moment-to-moment changes in viewing conditions, such as happens every time the eyes move. Together with previous human studies (Knill and Saunders, 2003; Oruç et al., 2003; Hillis et al., 2004; Welchman et al., 2005; Preston et al., 2009; Murphy et al., 2013; Rideaux and Welchman, 2018), our results suggest that reliability-dependent cue integration is a conserved computation underlying robust 3D vision in primates.

### Canonical computations might optimize but do not maximize 3D perception

Using simulations, we examined how neural network architectures and computations can constrain perception. Comparisons between the perceptual data and the simulation results produced testable predictions regarding where and how perception might be curbed by neuronal processing. The perceptual data suggested an architecture in which two independent populations representing stereoscopic cues and perspective cues (from both eyes) were optimally integrated. Thus, a critical question was how left and right eye perspective signals were combined by the perspective cue population. We found that the perceptual data could be explained if the two perspective signals were combined with quadratic nonlinearities and divisively normalized. These computations made the two representations fully dependent, precluding any improvement in perceptual precision that could have occurred from having both perspective cues. Humans show individual differences in the extent to which visual cues are dependent on each other (Oruç et al., 2003). It would be straightforward to incorporate such differences into the model. For example, down-weighting the normalization would result in partially dependent perspective cues, and thus increased perceptual precision. Since our focus was on computations specific to cue integration, and evidence for the influence of a prior was relatively small, we did not incorporate a prior over tilt into the model. If future work demonstrated sufficient need to incorporate a prior, there are several potential approaches to consider including heterogeneous tuning curves (Ganguli and Simoncelli, 2014) and tuned divisive normalization (Rosenberg et al., 2015). However, it is currently unclear how priors are physiologically implemented. Future work aimed at answering this question would be valuable for understanding neural computation.

Because of divisive normalization in the two independent populations model that accounted for our findings, the perceptual contributions of left and right eye perspective cues can range from averaging (both signals contribute equally) when they are equally reliable to winner-take-all (only the more reliable signal contributes) when they differ substantially (Busse et al., 2009). As such, this architecture can also explain human cue integration findings that show cue averaging with balanced perspective cues in slant perception (Knill and Saunders, 2003; Hillis et al., 2004), as well as winner-take-all behavior with imbalanced perspective cues in motion-in-depth perception (Thompson et al., 2019). Importantly, this model provides testable predictions about where and how neural computation limits tilt perception. One prediction is that if perspective cues were presented to both eyes without stereoscopic cues, then the response would be equal to that elicited from single-eye stimulation. Future neuronal recording experiments can test this by presenting random dot stimuli binocularly with appropriate perspective rendering for each eye but uncorrelated dot positions across the eyes.

Our simulations showed that independent representations of stereoscopic cues, left eye perspective cues, and right eye perspective cues could double the precision of tilt perception. Why then has the visual system not implemented this network and integration strategy? One possibility is the biological inefficiency associated with the number of neurons required to maintain three separate representations, and the duplication of computational units to separately estimate 3D information from left and right eye perspective signals. Another possibility is that 3D estimates derived from perspective cues are noisy, and pooling the two signals is necessary for noise reduction.

The current results are consistent with optimized (through the linear summation of independent stereoscopic cue and perspective cue population responses) but not maximized (due to combining left and right eye perspective cue representations using quadratic nonlinearities and divisive normalization) 3D perception. Analogous limitations may exist in other sensory systems which have multiple inputs sensitive to the same signals, as occurs in audition, vestibular processing, and bimanual touch. Lastly, our results highlight that “optimal” is in the eye of the beholder, and most meaningful in the context of a specific neural architecture.

## Synthesis

Reviewing Editor: Siegrid Löwel, University of Goettingen Decisions are customarily a result of the Reviewing Editor and the peer reviewers coming together and discussing their recommendations until a consensus is reached. When revisions are invited, a fact-based synthesis statement explaining their decision and outlining what is needed to prepare a revision will be listed below. The following reviewer(s) agreed to reveal their identity: Detlef Wegener.

This is an interesting study that test 3D vision in monkeys using psychophysics and use this data to put to the test a variety of models of cue integration. The main conclusion is that integration of visual cues for depth is optimal but alternative integration strategies would lead to even greater increments in precision.

In particular, the authors investigate 3D perception in non-human primates for a large variety of different tilt and slant conditions, at different distances. To do this, they use stimuli having either both stereoscopic and perspective cues to estimate orientation in depth, or have only one of the two cues. This enabled them to investigate to what extent each of the cues would contribute to perception. The authors also performed a variety of control experiments to check their results. Besides analysis of behavioral data the authors use modelling approaches to determine how 3D perception arises from stereoscopic and perspective cues. They found that perceptual data were most consistent with a model in which two independent neuronal populations combine stereoscopic and perspective cues and compare this to alternative models using three independent or only one population to process the depth cues.

The paper provides a comprehensive analysis of 3D perception in non-human primates. Particularly the model (using simulations of data from monkey caudal intraparietal area) provides new insights into how the visual system may integrate stereoscopic and perspective cues to process depth information. The research question is well introduced and the Methods section provides a detailed report on procedures and analyses including qualitative explanations to better inform the reader. Methods are rigid and reported in sufficient detail to allow for reproduction of the experiments. Results are easy to follow and provide an in-depth analysis of cue integration for 3D perception.

Overall, this is a nice paper: the experiments are properly conducted and the modeling section is highly informative and equally sound.

Nevertheless, we feel that taking this dataset to support using non-human primates as a valid model system for 3D cue integration seems a bit out of place. First of all because non-human primates, along with several other animal models (including insects) have already been used extensively. But more specifically, that would require a direct comparison of humans and non-human data using the same setup, stimuli and task. This would have been a great addition to this study, which only relies on data from two animals (though these datasets were in all fairness rich enough, consisting of several thousand trials).

Minor comments:

- Slant, tilt, and distance are the parameters the reader needs to understand to follow the data. Slant and distance are nicely introduced in Figure 1 and 2 but tilt is explained in text only. For the reader not too familiar with 3D stimuli it would be informative to visually introduce the tilt parameter as well, and how it combines with slant.

- In Methods, it is described that monkeys were rewarded for a saccade to the correct target. Given that there seemed to be quiet some conditions for which it was nearly impossible to indicate the correct target better than chance (e.g. for stereoscopic cues at larger distances) I wondered how the authors kept monkeys motivated and alert. Was the overall number of trials with chance-level performance small enough to not influence motivation to perform the task? Did it happen that monkeys tended to terminate such trials quite soon instead of waiting for the choice display? As far as I understood, such trials would not enter data analysis. Correct?

- L100: ... fixated on the --> ...fixating the (?)

## Author Response

Significance Statement Comments for Author (Required):

N/A

Comments on the Visual Abstract for Author (Required):

NA/

Synthesis Statement for Author (Required):

This is an interesting study that test 3D vision in monkeys using psychophysics and use this data to put to the test a variety of models of cue integration. The main conclusion is that integration of visual cues for depth is optimal but alternative integration strategies would lead to even greater increments in precision.

In particular, the authors investigate 3D perception in non-human primates for a large variety of different tilt and slant conditions, at different distances. To do this, they use stimuli having either both stereoscopic and perspective cues to estimate orientation in depth, or have only one of the two cues. This enabled them to investigate to what extent each of the cues would contribute to perception. The authors also performed a variety of control experiments to check their results. Besides analysis of behavioral data the authors use modelling approaches to determine how 3D perception arises from stereoscopic and perspective cues. They found that perceptual data were most consistent with a model in which two independent neuronal populations combine stereoscopic and perspective cues and compare this to alternative models using three independent or only one population to process the depth cues.

The paper provides a comprehensive analysis of 3D perception in non-human primates. Particularly the model (using simulations of data from monkey caudal intraparietal area) provides new insights into how the visual system may integrate stereoscopic and perspective cues to process depth information. The research question is well introduced and the Methods section provides a detailed report on procedures and analyses including qualitative explanations to better inform the reader. Methods are rigid and reported in sufficient detail to allow for reproduction of the experiments. Results are easy to follow and provide an in-depth analysis of cue integration for 3D perception.

Overall, this is a nice paper: the experiments are properly conducted and the modeling section is highly informative and equally sound.

Dear Dr. Löwel, we thank you and the reviewers for their thoughtful feedback on our manuscript. We are extremely pleased that the manuscript was so well received. In particular, we feel that clarifications regarding tilt and its relationship to slant, the effect of conditions with chance-level performance on the motivation of the monkeys, and the handling of aborted trials have much improved the manuscript.

Nevertheless, we feel that taking this dataset to support using non-human primates as a valid model system for 3D cue integration seems a bit out of place. First of all because non-human primates, along with several other animal models (including insects) have already been used extensively. But more specifically, that would require a direct comparison of humans and non-human data using the same setup, stimuli and task. This would have been a great addition to this study, which only relies on data from two animals (though these datasets were in all fairness rich enough, consisting of several thousand trials).

Thank you for pointing this out. We agree with this assessment. We strongly considered repeating the study with humans. Unfortunately, it would not be possible to use the same setup since the structure is built around a small monkey chair. We also cannot move the projector to another location since it requires substantial calibration if it is moved, and the setup is currently being used for other monkey experiments. Since the data set was considered rich enough without human data, we opted to remove the statement regarding validating the macaque as a model system for studying 3D cue integration from the Discussion. This edit is found on page 31, lines 926 - 927 of the manuscript with changes tracked.

Minor comments:

- Slant, tilt, and distance are the parameters the reader needs to understand to follow the data. Slant and distance are nicely introduced in Figure 1 and 2 but tilt is explained in text only. For the reader not too familiar with 3D stimuli it would be informative to visually introduce the tilt parameter as well, and how it combines with slant.

We completely agree that the manuscript would benefit from a visual introduction to tilt. We added a new panel (Figure 2A) to illustrate tilt, slant, and how the two variables combine. The new figure and appropriately edited figure legend is found on page 7 of the manuscript with changes tracked. Corresponding edits were made on: page 5, lines 170 - 175 of the manuscript with changes tracked.

- In Methods, it is described that monkeys were rewarded for a saccade to the correct target. Given that there seemed to be quiet some conditions for which it was nearly impossible to indicate the correct target better than chance (e.g. for stereoscopic cues at larger distances) I wondered how the authors kept monkeys motivated and alert. Was the overall number of trials with chance-level performance small enough to not influence motivation to perform the task? Did it happen that monkeys tended to terminate such trials quite soon instead of waiting for the choice display? As far as I understood, such trials would not enter data analysis. Correct?

These are all great points, which we should have made clearer.

We have added text which indicates that the number of stimulus conditions for which the monkeys performed at chance-level was low post training (~1 - 8%, depending on the monkey), and that this had no obvious effect on their motivation to perform the task. These edits are found on page 8, lines 243 - 246 of the manuscript with changes tracked.

We have also clarified that aborted trials were put back into the block at pseudo-random locations in the trial order. In this way, the analyses included a roughly equal number of trials for each stimulus condition. We also added statistical results which indicate that the percentage of aborted trials did not depend on the tilt, slant, distance, or cue condition. The lack of a relationship here likely reflects that the monkeys knew that they needed to respond to all of the stimuli before a block completed, thus there was no motivation to skip hard trials. These edits are found on page 8 and 9, lines 261 - 264 of the manuscript with changes tracked.

Yes, that is correct - the aborted trials were discarded in the analysis. We clarified this on page 8, lines 255 - 256 of the manuscript with changes tracked.

- L100: ... fixated on the --> ...fixating the (?)

This is now fixed, thank you.
